# Magnetic Nanoparticle-Assisted Immobilized Co-Culture of *Saccharomyces cerevisiae* and *Kluyveromyces marxianus*: Effects on Ethanol Production, Sugar Consumption, Biomass Reuse, and Volatile Metabolite Profiles

**DOI:** 10.3390/jof12080593

**Published:** 2026-08-10

**Authors:** Arianna Núñez-Caraballo, Rodolfo Ramos-González, Cristóbal N. Aguilar, Georgina Michelena-Álvarez, Miguel A. Aguilar-González, José L. Martínez-Hernández, Anna Iliná

**Affiliations:** 1 Nanobioscience Group, School of Chemistry, Universidad Autónoma de Coahuila, Saltillo 25280, Coahuila, Mexico; ariannanunez@uadec.edu.mx; 2 Secretaría de Ciencia, Humanidades, Tecnología e Innovación, Universidad Autónoma de Coahuila, Saltillo 25280, Coahuila, Mexico; rodolfo.ramos@uadec.edu.mx; 3 Group of Bioprocesses and Bioproducts, Food Research Department, Universidad Autónoma de Coahuila, Saltillo 25280, Coahuila, Mexico; cristobal.aguilar@uadec.edu.mx; 4 Instituto Cubano de Investigaciones de los Derivados de la Caña de Azúcar (ICIDCA), Ciudad de la Habana 11000, Cuba; georgina_michelena@yahoo.com.mx; 5 Center for Research and Advanced Studies of the National Polytechnic Institute (CINVESTAV-IPN), Unidad Saltillo, Ramos Arizpe 25900, Coahuila, Mexico; miguel.aguilar@cinvestav.edu.mx

**Keywords:** *Saccharomyces cerevisiae*, *Kluyveromyces marxianus*, co-culture fermentation, chitosan-coated magnetic nanoparticles, yeast interactions, ethanol production, volatile compounds, sugarcane molasses

## Abstract

Yeast-assisted mixed-culture fermentations have gained attention for their ability to enhance fermentation efficiency and modulate metabolite production. There is little knowledge on the impact of magnetic nanoparticle-assisted immobilization on yeast–yeast interactions during alcoholic fermentation. The co-culture platforms of *Saccharomyces cerevisiae* and *Kluyveromyces marxianus*, immobilized on chitosan-coated manganese ferrite nanoparticles, were applied in the fermentation of sugarcane molasses and sugarcane juice in the present study. The chitosan-coated MnFe_2_O_4_ nanoparticles were prepared using a one-step coprecipitation reaction followed by hydrothermal treatment and were characterized by X-ray diffraction, Fourier transform infrared spectroscopy, vibrating sample magnetometry, and scanning electron microscopy. An immobilized co-culture system has been shown to provide faster sugar consumption and higher ethanol production than non-immobilized-cell fermentations. Operational stability of the immobilized biomass and higher ethanol production by reuse were confirmed by repeated fermentation cycles. Bioproduction of volatile metabolites varies among monoculture, co-culture, non-immobilized-cell, and immobilized systems, with yeast interactions and magnetic immobilization also affecting secondary metabolite production during fermentation. The findings confirm that magnetic nanoparticle-assisted co-culture fermentation would be an attractive nanobiotechnological tool for enhancing alcoholic fermentation and shed new light on yeast interactions with the nanostructured system when combined with the immobilized solution.

## 1. Introduction

Alcoholic fermentation by yeast remains one of the key biotechnological processes used to produce drinks, biofuels, and value-added compounds [1]. Since these microorganisms are important for optimizing fermentation efficiency, substrate utilization, stress tolerance, and the generation of desirable metabolites, there has been growing interest in research on yeast interactions in mixed cultures over the past decade [2]. Mixed-culture fermentation has emerged not only in the conventional food and beverage industry but also for sustainable bioprocesses in the valorization of agro-industrial residues and renewable carbon sources [3].

Of all the fermentative yeasts, *Saccharomyces cerevisiae* is probably the most widely used microorganism for industrial ethanol production, as it demonstrates high fermentative capacity, tolerance to ethanol, osmotic resistance, and adaptability to industrial conditions [4]. However, non-*Saccharomyces* yeasts such as *Kluyveromyces marxianus* have garnered greater attention for their rapid growth, thermotolerance, substrate versatility, and ability to promote the formation of volatile and sensory-active substances during fermentation [5]. If these yeast species interact in co-culture systems, synergistic metabolic responses can occur, affecting ethanol production, sugar assimilation, and metabolite formation.

Co-culture fermentations have proven an effective approach to improving biotechnological performance by leveraging the physiological attributes of diverse organisms [3,6]. Microbial interactions in yeast mixed cultures might include nutrient competition, metabolite exchange, stress adaptation, quorum-like responses, and adjustments to the extracellular environment. Interactions of this nature can significantly influence the kinetics of fermentation and the production of secondary metabolites (higher alcohols, esters, aldehydes, and organic acids) that play crucial roles in the sensory quality of fermented products [3,6]. While mixed fermentations are receiving much research interest, there is limited knowledge about the impact of nanostructured immobilization systems on yeast–yeast interactions and metabolite production.

Cell immobilization approaches have been widely studied as alternatives to non-immobilized-cell fermentation systems because they offer simpler biomass recovery, increased operational stability, protection against inhibitory compounds, and the reusability of biocatalysts across successive fermentation runs [7,8]. Magnetic nanoparticles have recently attracted considerable interest as immobilization supports due to their large surface area, adsorption capacity, and ease of separation in external magnetic fields, leading to extensive development of these compounds in recent years [9,10]. Among these materials, manganese ferrite nanoparticles exhibit promising magnetic properties and have been identified for applications in biotechnology and microbial processes. Manganese ferrite nanoparticles coated with chitosan can retain biocompatibility and provide functional groups that interact with microbial cell surfaces [9,10].

In previous studies, *S. cerevisiae* immobilization on chitosan-coated manganese ferrite nanoparticles was found to enhance ethanol fermentation and reduce fermentation time in cane molasses [11]. Nonetheless, the influence of magnetic nanocomposite-aided immobilization on mixed yeast cultures and their co-mediated interactions remains poorly understood. Of particular significance, little is known about the effect of co-immobilization on fermentation efficiency, biomass reuse, and the formation of volatile metabolites.

Sugarcane molasses and sugarcane juice are appealing substrates for alcoholic fermentation because of their very high sugar content and their abundant availability as agro-industrial raw materials [12]. Efficient fermentation using these media and/or substrates is a significant step toward the valorization of sugar industry by-products, benefiting environmentally friendly bioeconomy systems [13]. Herein, the integration of hybrid yeast cultures with magnetic nanobiocatalytic systems offers promising prospects for enhancing fermentation and investigating microbial interactions in an immobilized environment.

Despite the growing interest in mixed-culture fermentations and magnetic nanoparticle-based immobilization systems, little information is available regarding how magnetic co-immobilization affects yeast–yeast interactions, fermentation performance, biomass reusability, and volatile metabolite production in sugarcane-derived substrates. Previous studies [11] have primarily focused on single-species immobilization systems, whereas the effects of magnetic nanoparticle-assisted co-cultures on fermentation kinetics and metabolite profiles remain poorly understood. Therefore, this study addresses an important knowledge gap by evaluating the performance of immobilized co-cultures of *Saccharomyces cerevisiae* and *Kluyveromyces marxianus* on chitosan-coated manganese ferrite nanoparticles and assessing their effects on ethanol production, sugar consumption, biomass reuse, and volatile metabolite formation.

Therefore, the aim of the present study was to investigate the impact of magnetic nanoparticle-enabled co-culture fermentation with *Saccharomyces cerevisiae* and *Kluyveromyces marxianus*, immobilized on chitosan-coated manganese ferrite nanoparticles, in sugarcane molasses and sugarcane juice. The effects of yeast interactions on ethanol production, sugar consumption kinetics, biomass reuse, and volatile metabolite formation, compared with non-immobilized-cell systems, have attracted particular attention.

## 2. Materials and Methods

### 2.1. Microorganisms and Inoculum Preparation

The yeast strains utilized in the co-culture system were *Saccharomyces cerevisiae* and *Kluyveromyces marxianus*. Both strains were obtained from the official culture collection of the Food Research Department at the Faculty of Chemical Sciences, Autonomous University of Coahuila (UAdeC, Saltillo, Mexico). *S. cerevisiae* is an industrial thermotolerant strain whose baseline performance and cultural conditions within our laboratory workflows were previously described by Núñez-Caraballo et al. [11], following the original industrial distillery assessments by Jover-de la Prida et al. [14]. The authentication and immobilization parameters for *K. marxianus* on magnetic nano-supports were previously reported. The strains were maintained at 4 °C on YPG agar medium containing glucose (20 g/L), peptone (10 g/L), yeast extract (10 g/L), and agar (15 g/L) from Merck México.

For inoculum preparation, a loopful of each yeast culture was transferred separately into 30 mL of sterile YPG medium and incubated at 32 °C for 18 h under agitation at 150 rpm until reaching approximately 10 ^7^ cells/mL. Cell concentration was determined using a Neubauer counting chamber (Hausser Scientific, Horsham, PA, USA) under optical microscopy. For co-culture experiments, equal volumes of each inoculum were mixed immediately before fermentation.

### 2.2. Raw Materials and Fermentation Medium

Sugarcane molasses and sugarcane juice obtained from the sugar industry of Tamaulipas, Mexico, were used as fermentation substrates. The molasses and juice were characterized according to standard analytical procedures [13]. Specifically, soluble solids were measured as °Brix using a digital refractometer Milwaukee MA871 (Milwaukee Instruments, Rocky Mount, NC, USA). The pH was determined using a calibrated digital pH meter. Fermentable and reducing sugars were quantified using the 3,5-dinitrosalicylic acid (DNS) method and validated by HPLC as described in Section 2.8. Total nitrogen content was quantified via the micro-Kjeldahl digestion and distillation method. The total ash content was determined gravimetrically by calcination in a muffle furnace until a constant weight was achieved. Crucially, the quantitative mineral profile of the resulting ashes (Mg, P, K, Ca, and S) was determined using X-ray Fluorescence (XRF) spectroscopy (Malvern Panalytical, Malvern, UK).

The fermentation medium was prepared by diluting molasses or sugarcane juice to 20 °Brix using distilled water supplemented with KH_2_PO_4_ (1.0 g/L), (NH_4_)_2_SO_4_ (1.59 g/L), and MgSO_4_·7H_2_O (0.5 g/L), all reagents from Merck, Mexico. The medium was sterilized by autoclaving prior to inoculation.

### 2.3. Synthesis of Chitosan-Coated Manganese Ferrite Nanoparticles

Chitosan-coated manganese ferrite nanoparticles were synthesized by one-step coprecipitation followed by hydrothermal treatment [15,16]. Ferric chloride hexahydrate and manganese sulfate were dissolved in distilled water at 70 °C to maintain an Fe: Mn molar ratio of 2:1. Medium-molecular-weight chitosan with a ~75% degree of deacetylation was added at 0.125% (*w*/*v*) under continuous stirring until fully solubilized. Manganese ferrite nanoparticles without chitosan were prepared under the same conditions.

Subsequently, sodium hydroxide solution (8 M) was added dropwise until pH 14 was reached. The reaction mixture was subjected to hydrothermal treatment in an autoclave for 5 h. The resulting dark brown precipitate was magnetically separated, washed repeatedly with distilled water until neutral pH, dried at 40 °C, and ground into powder.

### 2.4. Characterization of Magnetic Nanoparticles

The crystalline structure of the nanoparticles was evaluated by X-ray diffraction (XRD) using Cu-Kα radiation over the 2θ range of 10–80°. Crystallite size was estimated using the Scherrer equation.

Magnetic properties were determined using an alternating gradient magnetometer (AGM) at room temperature. Fourier transform infrared spectroscopy (FTIR, Thermo Nicolet Nexus, Madison, WI, USA) was performed in the range of 900–4000 cm^−1^ to verify the presence of chitosan functional groups on the nanoparticle surface [9,10].

The chitosan-coated manganese ferrite nanoparticles employed as immobilization supports were prepared according to previously reported procedures [16]. Their physicochemical, morphological, particle-size, and magnetic characteristics, including analyses by HRTEM, TGA, TEM, and DLS, were previously reported [16]. Therefore, the present study focused on evaluating their application in yeast co-immobilization and alcoholic fermentation.

### 2.5. Yeast Immobilization on Magnetic Nanoparticles

Immobilization assays were performed by mixing yeast suspensions with chitosan-coated manganese ferrite nanoparticles at a final concentration of 460 mg/mL. The mixtures were incubated for 30 min under agitation at room temperature. Immobilized cells were separated under an external magnetic field. Quantifying non-immobilized cells remaining in the supernatant using a Neubauer chamber confirmed complete cell immobilization; no free cells were observed by Neubauer counting. The immobilized biomass was subsequently used in fermentation experiments.

The morphology of the non-immobilized yeasts and cell-nanoparticle complexes was evaluated using scanning electron microscopy (SEM) as described by Núñez-Caraballo et al. [11]. Prior to analysis, samples were lyophilized, dispersed in absolute ethanol, fixed onto a polished bronze mirror holder, and sputter-coated with copper (99.97% purity) in a high-vacuum evaporator (JEOL, JEE400 JEOL Ltd., Tokyo, Japan) at 2 × 10^−5^ mbar and 18 mA for 15 s. Micrographs were acquired using an environmental scanning electron microscope (ESEM, Philips XL-30, Cambridge, UK) operated at an accelerating voltage of 20–40 keV, a spot size of 4.5, and a working distance of 39 mm.

### 2.6. Alcoholic Fermentation Assays

Alcoholic fermentation experiments were carried out in 500 mL Erlenmeyer flasks containing 300 mL of fermentation medium. Initially, two experimental systems were evaluated: non-immobilized co-culture and immobilized co-culture of *S. cerevisiae* and *K. marxianus*. The inoculum concentration was approximately 1 × 10^−6^ cells/mL. To establish anaerobic conditions and manage gas release, the flasks were hermetically sealed with rubber caps and equipped with glass capillaries connected to a CO_2_ trap (airlock system), as previously specified by Núñez-Caraballo et al. [11]. Fermentations were performed at 32 °C and 150 rpm for 42 h. Samples were collected periodically for analysis of sugars and ethanol production.

### 2.7. Reuse of Immobilized Biomass

The immobilized co-culture system underwent repeated fermentation cycles to assess biomass reuse. After each fermentation cycle, the immobilized biomass was recovered by applying an external magnetic field, washed with sterile distilled water, and transferred to a fresh fermentation medium containing molasses under the same operational conditions.

### 2.8. Quantification of Sugars and Ethanol

Glucose, fructose, and ethanol concentrations were quantified by high-performance liquid chromatography (HPLC) using a Waters system (Waters Corp., Milford, MA, USA) equipped with a model 600E quaternary pump, a model 717 automatic injector, and a model 410 refractive index detector. Chromatographic separation was achieved using an Aminex HPX-87P column (300 × 7.8 mm, Bio-Rad, Hercules, CA, USA). The mobile phase consisted of 5 mM H_2_SO_4_ at a flow rate of 0.65 mL/min. The column temperature was maintained at 60 °C, and the injection volume was 20 µL. Data acquisition and processing were performed using Empower 3 software.

### 2.9. Determination of Volatile Compounds

Fermented molasses-containing media were subjected to distillation using a laboratory distillation system. A volume of 250 mL of fermented must was collected in order to avoid dragging sedimented yeast cells from the bottom of the fermentation flask. The fermented broth was transferred into a 500 mL round-bottom flask and heated using a heating mantle or hot plate. The vapor temperature was continuously monitored at 80 °C with a thermometer attached to the flask neck. The generated vapors passed through a condenser, where they were cooled, and the condensate was collected in a graduated cylinder until the final volume reached exactly 50 mL of distillate for all treatments, ensuring a uniform concentration factor for subsequent analytical comparison. Heat exchange inside the condenser occurred between the vapors released from the fermented broth and chilled water. After completion of the distillation process, the ethanol-depleted residue (vinasse) was discarded.

The major volatile compounds present in the distillates were quantified using standard analytical methods and expressed in g/100 L of alcohol. The distillates obtained from the different fermentation assays (with non-immobilized and immobilized *S. cerevisiae*; non-immobilized *K. marxianus*; non-immobilized and immobilized co-culture of *S. cerevisiae* and *K. marxianus*) were analyzed by gas chromatography. A gas chromatograph Varian (model CP-3800, Palo Alto, CA, USA) equipped with a Flame Ionization Detector (FID) was used for the analyses. Samples of 1 µL of distillates and standards were injected in split mode with a split ratio of 1:20. The analyses were performed using a CP-WAX 10 capillary column (50 m × 0.32 mm internal diameter, 0.2 µm film thickness). Ultra-high-purity helium was used as the carrier gas at a constant pressure of 10 psi and a flow rate of 1.5 mL/min. The column temperature program was as follows: 40 °C for 10 min, followed by heating from 40 °C to 70 °C at 5 °C/min. The injector temperature was maintained at 150 °C, and the FID temperature was kept at 250 °C. Peak identification was achieved by comparing retention times with those obtained from authentic external standards analyzed under identical chromatographic conditions. Quantification was based on chromatographic peak areas and calibration curves generated from the corresponding standards.

### 2.10. Statistical Analysis

All fermentation kinetics, volatile compound assays, and proximate raw material characterizations were performed in triplicate, and the results are expressed as mean ± standard deviation. Statistical analysis of the fermentation and volatile metabolite data was conducted using analysis of variance (ANOVA) at a significance level of *p* < 0.01. For the proximate characterization, significant differences between the two substrates were evaluated using Student’s *t*-test (*p* < 0.05). Furthermore, data for the elemental mineral profile represent absolute percentage values obtained from a high-precision, single-run X-ray Fluorescence (XRF) analysis of homogenized composite ash samples. All statistical analyses and differences between treatments were analyzed using Statistica 7.0 software (StatSoft, Tulsa, OK, USA).

## 3. Results

### 3.1. Characterization of Chitosan-Coated Manganese Ferrite Nanoparticles

The one-step coprecipitation method, followed by hydrothermal treatment, enabled the synthesis of chitosan-coated manganese ferrite nanoparticles with magnetic properties. The obtained material appeared as a dark brown powder that responded to an external magnetic field.

**Figure 1:**
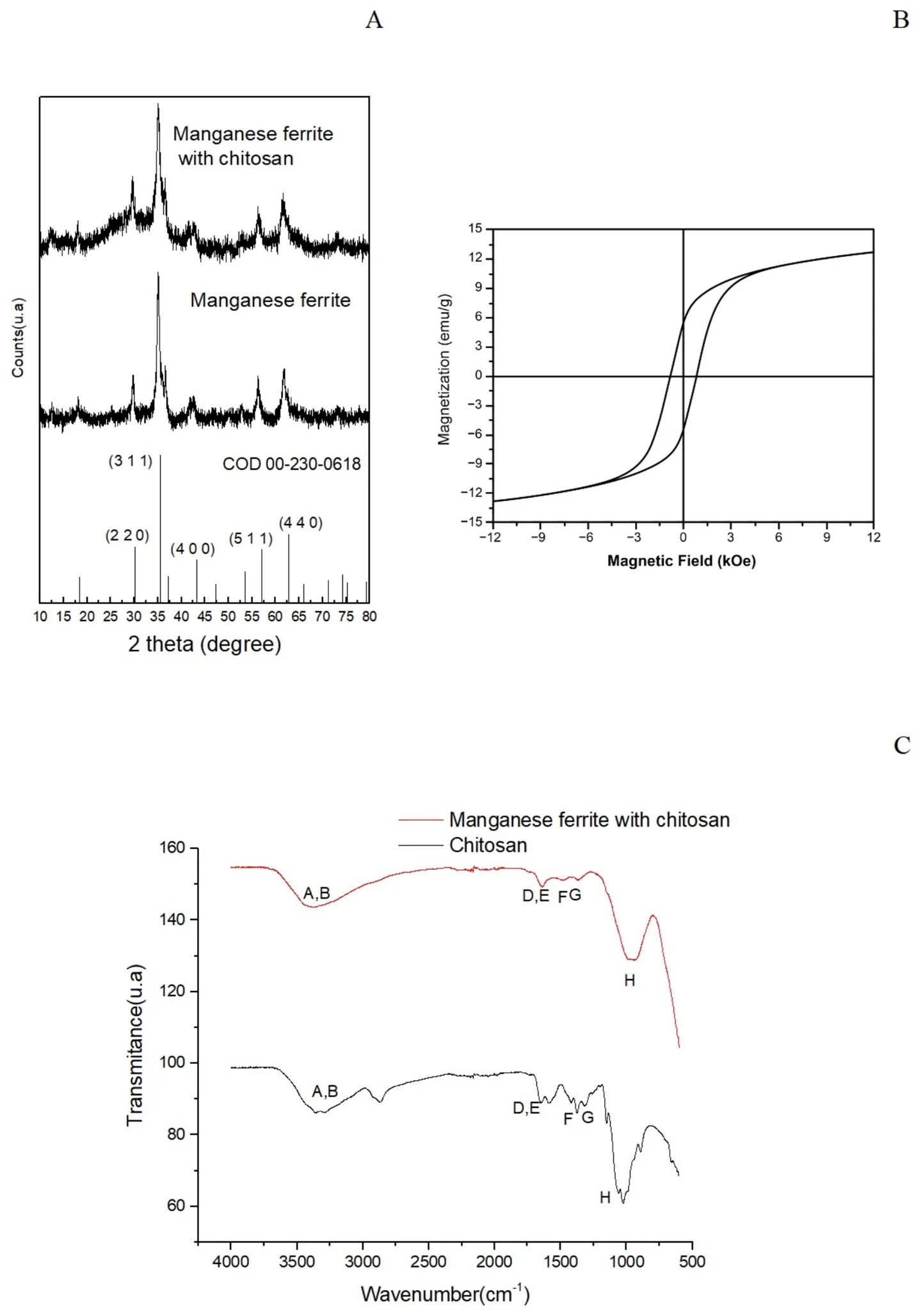
Physicochemical characterization of chitosan-coated manganese ferrite nanoparticles used for yeast immobilization. (**A**) X-ray diffraction (XRD) patterns of manganese ferrite nanoparticles synthesized with and without chitosan coating, showing the characteristic diffraction planes of MnFe_2_O_4_ spinel structure. (**B**) Magnetization curves obtained at room temperature for chitosan-coated manganese ferrite nanoparticles. (**C**) FTIR spectra of chitosan and chitosan-coated manganese ferrite nanoparticles, showing characteristic functional groups associated with chitosan coating. The presence of chitosan was confirmed by the appearance of characteristic bands near 3370 cm^−1^ (O-H and N-H stretching vibrations [A]), and 2865–2920 cm^−1^ (C–H stretching) [B], 1625–1660 cm^−1^ (N-H bending vibrations [D, E]), 1377–1400 cm^−1^ (C-N stretching vibrations [F, G]), and 1060–1061 cm^−1^ (C-O-C stretching vibrations [H]).

The X-ray diffraction patterns confirmed the formation of MnFe_2_O_4_ nanoparticles with a cubic spinel structure (Figure 1A). Characteristic diffraction peaks corresponding to the crystallographic planes (220), (311), (400), (511), and (440) were observed. The crystallite size determined by the Scherrer equation was 15.2 ± 1.02 nm for MnFe_2_O_4_ and 11.1 ± 0.07 nm for chitosan-coated MnFe_2_O_4_. Chitosan-coated manganese ferrite nanoparticles exhibited a smaller crystallite size compared with ferrite synthesized without chitosan coating.

Magnetic characterization revealed that the chitosan-coated manganese ferrite nanoparticles exhibited ferromagnetic behavior at room temperature, as evidenced by the presence of a hysteresis loop in the magnetization curve (Figure 1B). The nanoparticles showed a saturation magnetization (Ms) of 12.78 ± 0.43 emu/g, a coercive field (Hc) of 814.75 ± 1.74 Oe, and a remanent magnetization (Mr) of 3.87 ± 0.26 emu/g. These magnetic parameters indicate that the nanoparticles retained sufficient magnetic responsiveness after chitosan coating, enabling efficient recovery and reuse via external magnetic separation.

FTIR spectra demonstrated the presence of characteristic chitosan functional groups on the nanoparticle surface (Figure 1C). Bands corresponding to O–H, N–H, C–N, and C–O vibrations were detected, confirming successful chitosan coating of manganese ferrite.

### 3.2. SEM Analysis of Immobilized Yeasts

**Figure 2:**
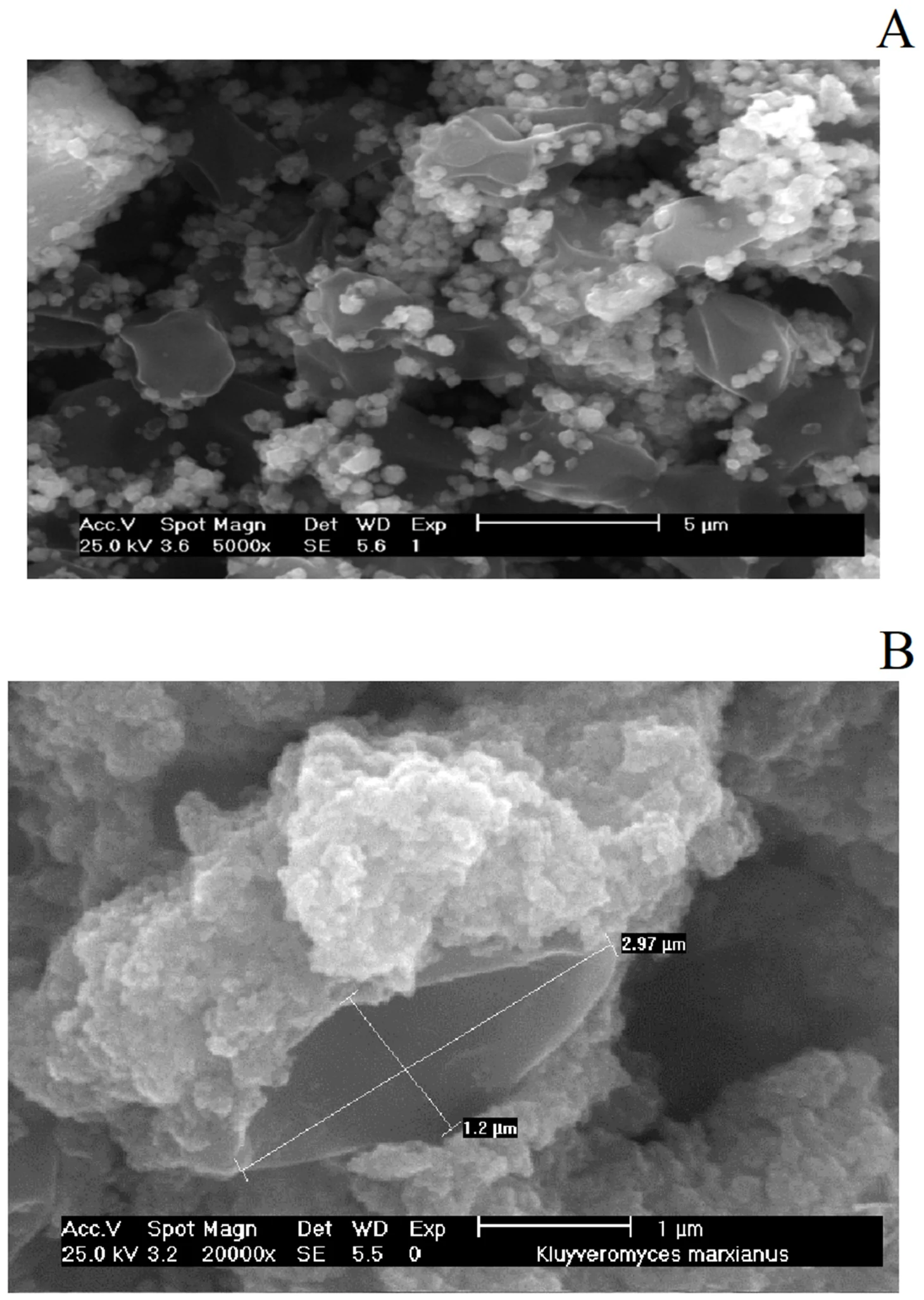
Scanning electron microscopy (SEM) micrographs of yeast immobilization on chitosan-coated manganese ferrite nanoparticles: (**A**) *Saccharomyces cerevisiae* and (**B**) *Kluyveromyces marxianus*. Detailed SEM image showing yeast interaction with aggregates of chitosan-coated manganese ferrite nanoparticles attached to the cell surface.

Figure 2 shows SEM micrographs of *S. cerevisiae* and *K. marxianus* on chitosan-coated manganese ferrite nanoparticles. Non-immobilized yeast cells showed the typical oval morphology characteristic of yeasts, with cell diameters ranging from approximately 2 to 4 µm. Upon immobilization, the cell surfaces appeared partially surrounded and covered by aggregates of magnetic nanoparticles, indicating close interaction with the chitosan-coated ferrite support.

The SEM images also showed the formation of compact nanoparticle clusters attached to the yeast cell walls, indicating the successful attachment and immobilization of yeast species on the matrix. There was no significant morphological damage or deformation of cells following immobilization, suggesting that the immobilization process preserved cellular integrity. Efficient magnetic recovery of the biomass further confirmed the successful attachment of yeast cells to the manganese ferrite nanoparticles, supporting their potential use in reusable fermentation systems.

The SEM micrographs confirmed the attachment of both yeasts to the nano-supports. Due to the separate immobilization protocol carried out to guarantee strict control over the initial cell ratios and avoid competitive surface adsorption, Figure 2A highlights the larger, spherical morphology characteristic of *S. cerevisiae* adhering to the C-MNPs, while Figure 2B displays the smaller, ellipsoidal structure of *K. marxianus* independently interacting with the matrix.

### 3.3. Sugar Consumption and Ethanol Production During Fermentation

Before fermentation, the substrates showed clear differences in composition (Table 1). Molasses presented much higher soluble solids (82.16 °Brix), fermentable sugars (66.34%), ash content (9.17%), and nitrogen (0.21%) than sugarcane juice, which contained 17.51 °Brix, 25.03% fermentable sugars, only 0.19% ashes, and no detectable nitrogen. This indicates that molasses is a more concentrated substrate, rich in sugars and minerals, whereas sugarcane juice is more dilute and less nutrient-dense for microbial growth. The pH of molasses was also lower (5.23) than that of sugarcane juice (3.51), which may favor yeast adaptation during fermentation.

The ash mineral profile (Table 2) confirmed that molasses contained higher potassium (0.09%) than sugarcane juice (0.03%), while calcium was similar in both substrates (0.03%). Magnesium was detected only in molasses, whereas phosphorus and sulfur were present at low and similar levels. Therefore, molasses may provide not only fermentable sugars but also mineral nutrients that can support alcoholic fermentation.

**Figure 3:**
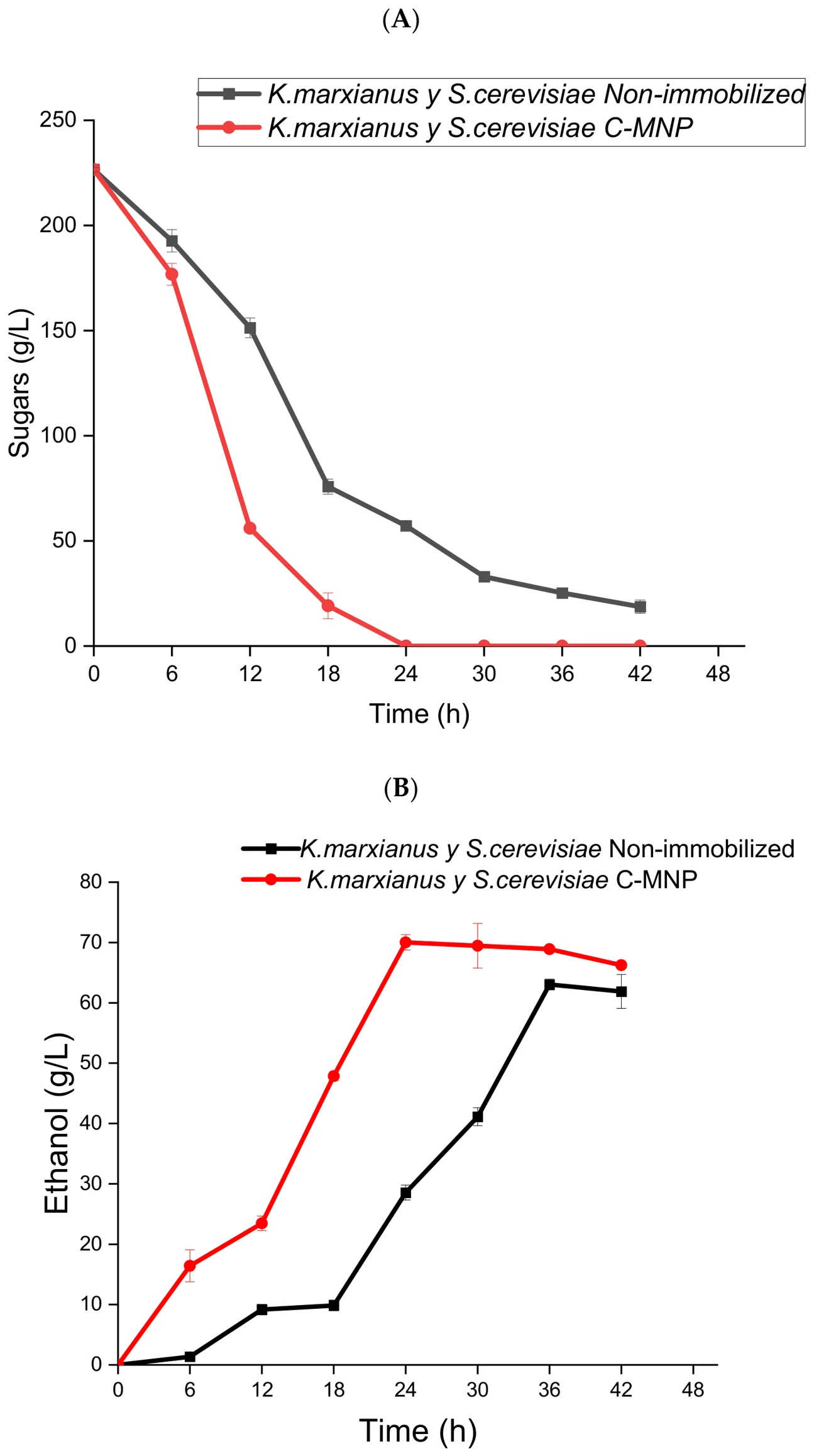
Fermentation kinetics of non-immobilized and immobilized co-culture yeast systems during sugarcane molasses fermentation at 20 °Brix and 32 °C. (**A**) Consumption of fermentable sugars by non-immobilized co-culture of *K. marxianus* and *Saccharomyces cerevisiae*, and co-immobilized yeasts on chitosan-coated manganese ferrite nanoparticles. (**B**) Ethanol production by the corresponding fermentation systems. Values represent mean ± standard deviation of triplicate experiments. In some cases, error bars are smaller than the symbols and may not be visible.

**Figure 4:**
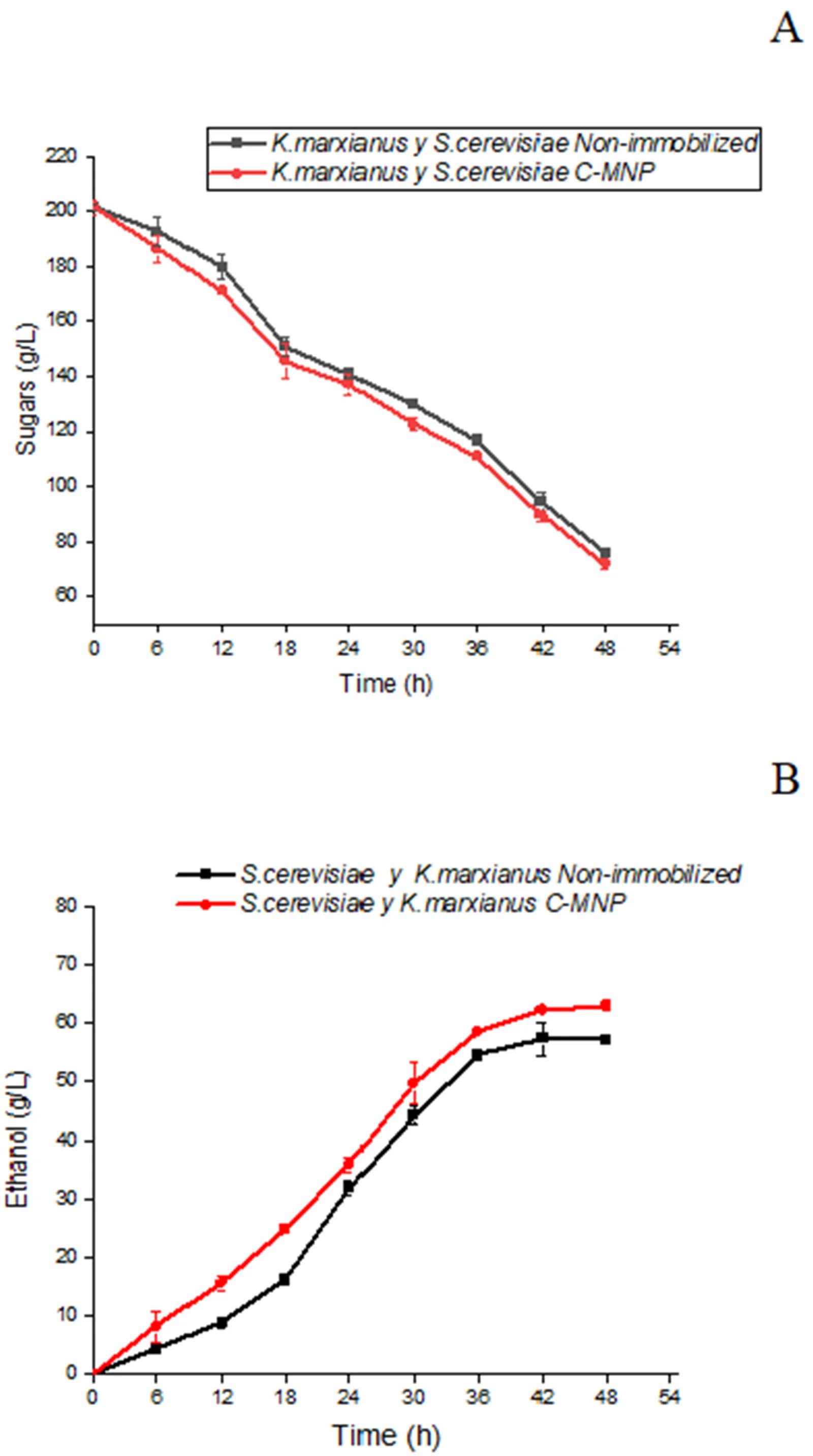
Fermentation kinetics of non-immobilized and immobilized co-culture yeast systems during sugarcane juice fermentation at 20 °Brix and 32 °C. (**A**) Consumption of fermentable sugars by non-immobilized co-culture of *K. marxianus* and *Saccharomyces cerevisiae*, and co-immobilized yeasts on chitosan-coated manganese ferrite nanoparticles. (**B**) Ethanol production by the corresponding fermentation systems. Values represent mean ± standard deviation of triplicate experiments. In some cases, error bars are smaller than the symbols and may not be visible.

The kinetics of sugar consumption and ethanol production during fermentation are shown in Figure 3 and Figure 4. The co-culture systems comprising *Saccharomyces cerevisiae* and *Kluyveromyces marxianus* exhibited rapid sugar assimilation in both molasses and sugarcane juice fermentations (Figure 3A and Figure 4A). Immobilized mixed cultures exhibited lower residual sugar concentrations than non-immobilized-cell co-cultures.

Ethanol production kinetics are presented in Figure 3B and Figure 4B. Immobilized yeast systems produced higher ethanol concentrations than non-immobilized-cell fermentations. So, the co-culture systems containing *S. cerevisiae* and *K. marxianus* showed enhanced ethanol production under immobilized conditions. In both molasses and sugarcane juice fermentations, immobilized co-cultures exhibited higher ethanol concentrations compared with non-immobilized mixed cultures (Figure 3B and Figure 4B).

These results indicate that magnetic nanoparticle-assisted immobilization enhanced fermentation performance by accelerating sugar utilization and promoting earlier ethanol accumulation compared with the non-immobilized co-culture system. The effect was particularly evident during molasses fermentation, where complete sugar consumption was achieved more rapidly in the immobilized treatment.

### 3.4. Reuse of Immobilized Biomass

The immobilized co-culture of *Saccharomyces cerevisiae* and *Kluyveromyces marxianus* demonstrated good operational stability during successive reuse cycles in sugarcane molasses fermentation. Ethanol production remained relatively constant during the first five cycles, with values close to 60–63 g/L, indicating that the immobilized biomass maintained high fermentative activity after magnetic recovery and reutilization. A slight, gradual decrease in ethanol production was observed after the fourth and fifth cycles, while a more pronounced reduction occurred in the sixth cycle, with the ethanol concentration decreasing to approximately 54 g/L. Despite this decline, the immobilized co-culture preserved a high percentage of its initial activity, confirming the effectiveness of chitosan-coated manganese ferrite nanoparticles as a support for cell immobilization and magnetic separation.

### 3.5. Volatile Compound Production in Different Fermentation Systems

The concentrations of major volatile compounds detected in the distillates of molasses-containing medium obtained after 42 h are presented in Table 3. Differences in volatile metabolite production were observed among monoculture, co-culture, non-immobilized-cell, and immobilized fermentation systems. The mixed cultures generated distinct volatile profiles compared with monoculture fermentations. Variations in concentrations of major volatile compounds were also observed between non-immobilized and immobilized systems.

Table 3 shows significant differences in volatile compound formation and ethanol production among the yeast systems studied during molasses fermentation. Among the monocultures, *K. marxianus* produced the lowest ethanol concentration (29.1 g/L) but generated the highest levels of ethyl acetate (3.54 g/100 L) and higher alcohols (145.0 g/100 L), indicating a greater tendency toward aroma compound formation. In contrast, *S. cerevisiae* 150 showed significantly higher ethanol production (50.7 g/L) with lower concentrations of volatile by-products, suggesting a more efficient conversion of sugars into ethanol. Significant differences (*p* < 0.01) were observed among fermentation systems for ethanol, ethyl acetate, acetaldehyde, and higher alcohol production.

The immobilization of *S. cerevisiae* 150 on chitosan-coated magnetic nanoparticles (C-MNPs) slightly improved ethanol production to 56.1 g/L and increased higher alcohol production to 83.02 g/100 L, while maintaining low acetaldehyde and methanol levels. These results indicate that immobilization positively influenced fermentative performance without substantially increasing undesirable volatile compounds (Table 3).

The co-culture systems exhibited the best fermentative performance. The combination of *K. marxianus* and *S. cerevisiae* 150 produced 63.1 g/L ethanol, together with the highest acetaldehyde concentration (15.37 g/100 L) and elevated higher alcohol production (180.0 g/100 L). This suggests a synergistic metabolic interaction between both yeasts that enhanced substrate utilization and secondary metabolite formation (Table 3).

The highest ethanol production was obtained with the immobilized co-culture of *K. marxianus* and *S. cerevisiae* 150 on C-MNPs, reaching 70.0 g/L. This system also produced the highest ethyl acetate concentration (4.77 g/100 L) and the greatest level of higher alcohols (185.4 g/100 L), while acetaldehyde levels were considerably lower than in the non-immobilized co-culture. These results demonstrate that immobilization improved fermentative efficiency and maintained active metabolic interactions between both yeast species (Table 3).

Overall, the results indicate that co-cultivation and magnetic immobilization positively affected ethanol productivity and volatile compound formation, suggesting that these strategies may enhance both fermentation performance and the aromatic profile of fermented products.

## 4. Discussion

### 4.1. Characterization of Magnetic Nanoparticles and Yeasts Immobilization

The XRD results verified that manganese ferrite nanoparticles have a cubic spinel crystalline structure, as evidenced by the characteristic diffraction peaks in the planes (Figure 1A). These diffraction patterns are in accord with the established MnFe_2_O_4_ spinel ferrites prepared with coprecipitation techniques [16], which suggest well-defined ferrite phase formation and no major secondary crystalline impurities. The reduction in crystallite size from 15.2 nm to 11.1 nm upon chitosan functionalization is chemically driven by the strong coordination of its primary amine (-NH_2_) and hydroxyl (-OH) groups with the Fe^3+^ and Mn^2+^ cations during the early stages of coprecipitation. This chelation effectively reduces the concentration of free metal ions available for rapid growth, promoting a higher initial nucleation rate (generating a larger population of smaller nuclei). Simultaneously, the adsorbed biopolymer chains form a steric and electrostatic capping barrier on the nascent crystal nuclei, which restricts the diffusion of solute ions toward the growing crystal facets during hydrothermal treatment, thereby suppressing subsequent grain growth [9]. A smaller crystallite size will increase surface area, which may be favorable for cell immobilization, as it exposes more interaction sites [17].

FTIR analysis also corroborated the coating of manganese ferrite nanoparticles with chitosan through characteristic bands found in O–H, N–H, C–N, and C–O functional groups (Figure 1C). The inclusion of chitosan is of great significance for immobilization, as the amino and hydroxyl groups in its structure enhance electrostatic interactions, hydrogen bonding, and surface adhesion between the support and microbial cells [18]. Moreover, chitosan is known for its biocompatibility, biodegradability, and low toxicity, making it an attractive candidate for biotechnological and fermentation applications. Adsorption of chitosan onto the nanoparticle surface may also promote colloidal stability and prevent excessive nanoparticle aggregation in aqueous media [19].

The synthesis of chitosan-coated manganese ferrite nanoparticles involves coordination chemistry, in which lone pairs on the amino (-NH_2_) and hydroxyl (-OH) groups of chitosan act as Lewis bases to coordinate to vacant d orbitals of surface metal cations (Fe^3+^ and Mn^2+^). Yeast immobilization is subsequently driven by electrostatic complementarity; at the operational pH, protonated amino groups (-NH^3+^) provide a positive surface charge that binds firmly to the net negative charge of the yeast cell walls (imparted by phosphomannans and carboxyl groups). This primary thermodynamic attraction is further stabilized by secondary hydrogen bonding between the cell envelope polysaccharides and the chitosan shell.

For the magnetic characterization, the chitosan-coated nanoparticles exhibited persistent ferromagnetic behavior upon functionalization (Figure 1B). However, the saturation magnetization was lower than that reported for uncoated ferrite nanoparticles [16,20]. This decrease is due to the nonmagnetic chitosan layer surrounding the magnetic core and the smaller crystallite size resulting from the coating process. On the other hand, the measured magnetic parameters were sufficient to achieve a fast magnetic response and effective separation under the influence of an external magnetic field. Preservation of magnetic characteristics after coating is important for practical fermentation, as the process enables easy recovery of biomass after immobilization, recycling, and isolation without centrifugation or filtration [18].

SEM data (Figure 2) also supported successful immobilization of yeast on the magnetic support. Both *S. cerevisiae* and *K. marxianus* retained their characteristic oval shape post-immobilization, thus showing that the immobilization process did not cause severe structural damage. The high degree of nanoparticle aggregation surrounding the yeast cells indicates strong interactions between the chitosan-coated ferrite surface and the microbial cell wall. The presence of polysaccharides, proteins, and negatively charged groups on the yeast surface that can interact with chitosan functional groups may facilitate this interaction [18]. When packed, nanoparticle clusters are grown on the cells, reflecting stable immobilization structures that retain biomass during fermentation. The lack of substantial cell deformation indicates that the immobilization conditions were mild and consistent with maintaining cellular viability and metabolic activity [15]. This is of particular importance for fermentation, in which cell integrity is required to maintain ethanol production and metabolite synthesis, in which preservation of cell structure is paramount [21]. In addition, the experimentally observed high magnetic recovery indicates that the immobilized biocatalyst can be readily separated and reused, which may benefit repeated-batch fermentation systems or industrial bioprocesses seeking to decrease operating costs and improve sustainability.

A more comprehensive physicochemical characterization of the nanostructured support, including specific surface area, zeta potential, particle size distribution, and colloidal stability measurements, may provide further insight into cell–material interactions and represents an important topic for future investigation.

### 4.2. Sugar Consumption and Ethanol Production During Fermentation

The compositional differences between molasses and sugarcane juice (Table 1 and Table 2) indicate that, although both substrates contain fermentable sugars, their nutritional profiles are markedly different and may affect yeast metabolism and subsequent fermentation efficiency. Molasses contains concentrations of sugars, minerals, ash, and nitrogen far greater than sugarcane juice, indicating that it may be a more favorable environment for yeast growth and ethanol formation. Nevertheless, the higher ash and mineral contents might cause osmotic stress, and cellular metabolism is especially affected by the high sugar concentration [13]. The low nitrogen content in the substrates, particularly sugarcane juice, suggests that the nitrogen available for assimilation is limited in fermentation. Nitrogen is critical for protein synthesis and enzyme production, cell growth, and the synthesis of key metabolites of alcoholic fermentation. Reduced nitrogen availability can lead to reduced yeast viability, lower fermentation rates, and increased production of off-target metabolites such as acetaldehyde and higher alcohols. Hence, supplementation with a nitrogen source and mineral salts may be required to maintain sufficient metabolic activity and fermentative capacity [11]. Furthermore, while direct tracking of population growth kinetics was restricted by the opacity of the sugarcane molasses and the magnetic supports, the operational phases can be effectively deduced from the metabolic profiles. When the system transitions into the stationary phase and experiences nutrient depletion (after 24 h), non-immobilized cells generally suffer from severe ethanol toxicity and metabolic exhaustion. However, the immobilized co-culture shifts into a highly stable, non-dividing biocatalytic state. The C-MNP matrix minimizes cell lysis under starvation conditions and acts as a physical shield against high ethanol concentrations, maintaining high cell viability and metabolic resilience at the end of the batch process [11].

Fermentation kinetics (Figure 3 and Figure 4) revealed that co-cultures of *Saccharomyces cerevisiae* and *Kluyveromyces marxianus* achieved complete sugar utilization. In both molasses and sugarcane juice fermentations, sugar assimilation was pronounced, suggesting vigorous metabolic cooperation among the yeast species. This duality could be related to the combined physiology of the two microorganisms. In particular, *K. marxianus* is known for its fast growth rate and broad substrate-assimilation capacity, and *S. cerevisiae* has high ethanol productivity and tolerance to fermentation-stress conditions [22]. The co-accumulation of both species may have facilitated more efficient substrate conversion during co-culture fermentation.

The low residual sugar content observed in immobilized mixed systems indicates improved fermentative activity in the study (Figure 3A and Figure 4A). Chitosan-coated manganese ferrite nanoparticles likely provided a suitable microenvironment that helped maintain cells, limit cell loss, increase overall cell density, and prolong metabolic activity [21]. Moreover, immobilization can lessen cell washout, enhance microbial contact with the fermentation medium, and increase substrate uptake and conversion efficiency [21]. Stable yeast systems, however, improve sugar uptake, which, due to increased mass transfer and environmental protection, supports improved fermentation performance.

Ethanol production profiles (Figure 3B and Figure 4B) also supported the beneficial impact of immobilization on fermentation efficiency. In both substrates, immobilized systems yielded higher ethanol concentrations than non-immobilized-cell fermentation in all experiments. These data demonstrate that magnetic support not only maintained cell viability but also supported optimal ethanol biosynthesis pathways. The higher ethanol yields in the immobilized co-cultures might also be attributed to increased metabolic stability, greater biomass retention, and protection of cells against inhibitory fermentation activity (e.g., ethanol accumulation, osmotic stress, or nutrient starvation) [21]. It has been shown that enhanced metabolic cooperation and substrate conversion pathways between *S. cerevisiae* and *K. marxianus* are observed in immobilized co-cultures compared with non-immobilized mixed cultures, owing to the immobilization matrix (Figure 3B and Figure 4B). Adding chitosan to the nanoparticle surface could also help preserve cell viability in a biocompatible environment, allowing for better fermentation over longer periods [15]. Individually or together, this would explain the elevated ethanol concentrations that we observed in this immobilized state. The above-mentioned results show that co-cultivation and magnetic immobilization together represent an excellent approach to improving alcoholic fermentation performance in sugar-rich substrates such as molasses and sugarcane juice. Furthermore, as observed in the immobilized systems, improved sugar levels, ethanol yields, and stable operating conditions indicated the suitability of these mechanisms for reusable, cost-effective bioethanol production. The enhanced fermentation performance observed in the immobilized co-culture may be associated with the characteristics of the chitosan-coated manganese ferrite support. Chitosan provides a biocompatible surface that facilitates cell attachment, while the magnetic ferrite core enables rapid biomass recovery and reuse. These features may improve cell retention within the system, thereby reducing biomass losses during repeated fermentation cycles and supporting ethanol productivity. Although additional physicochemical parameters such as specific surface area, zeta potential, particle size distribution, and colloidal stability were not evaluated in the present work, the results suggest that the nanostructured support provided a suitable microenvironment for maintaining fermentation performance during repeated use.

Beyond the statistically significant differences observed among treatments, the results revealed consistent trends indicating that immobilization positively influenced the evaluated fermentation parameters. The enhanced sugar consumption and ethanol production achieved by the immobilized co-culture may be attributed to the synergistic interaction between *S. cerevisiae* and *K. marxianus*, combined with the favorable microenvironment provided by the chitosan-coated magnetic nanoparticles. Such a microenvironment can facilitate cell retention, improve metabolic stability, and reduce biomass loss during repeated fermentation cycles, ultimately contributing to the superior performance of the immobilized system compared with free-cell fermentations.

The observed improvements in sugar assimilation, ethanol production, and volatile metabolite formation may be directly related to the physicochemical characteristics of the chitosan-coated manganese ferrite nanoparticles [21]. The reduced crystallite size and the presence of chitosan functional groups provide a favorable surface for yeast attachment and cell retention, facilitating close contact between cells and the fermentation medium. In addition, the magnetic properties of the support enable efficient biomass recovery while maintaining an immobilized microenvironment that may reduce cell loss and enhance metabolic stability [16,19,21]. These factors likely contributed to the accelerated sugar consumption and increased ethanol production observed in the immobilized systems [21]. Furthermore, the modified microenvironment generated by immobilization may influence metabolic fluxes associated with secondary metabolite biosynthesis, contributing to the differences observed in volatile compound profiles between immobilized and free-cell fermentations.

### 4.3. Reuse of Immobilized Biomass

**Figure 5:**
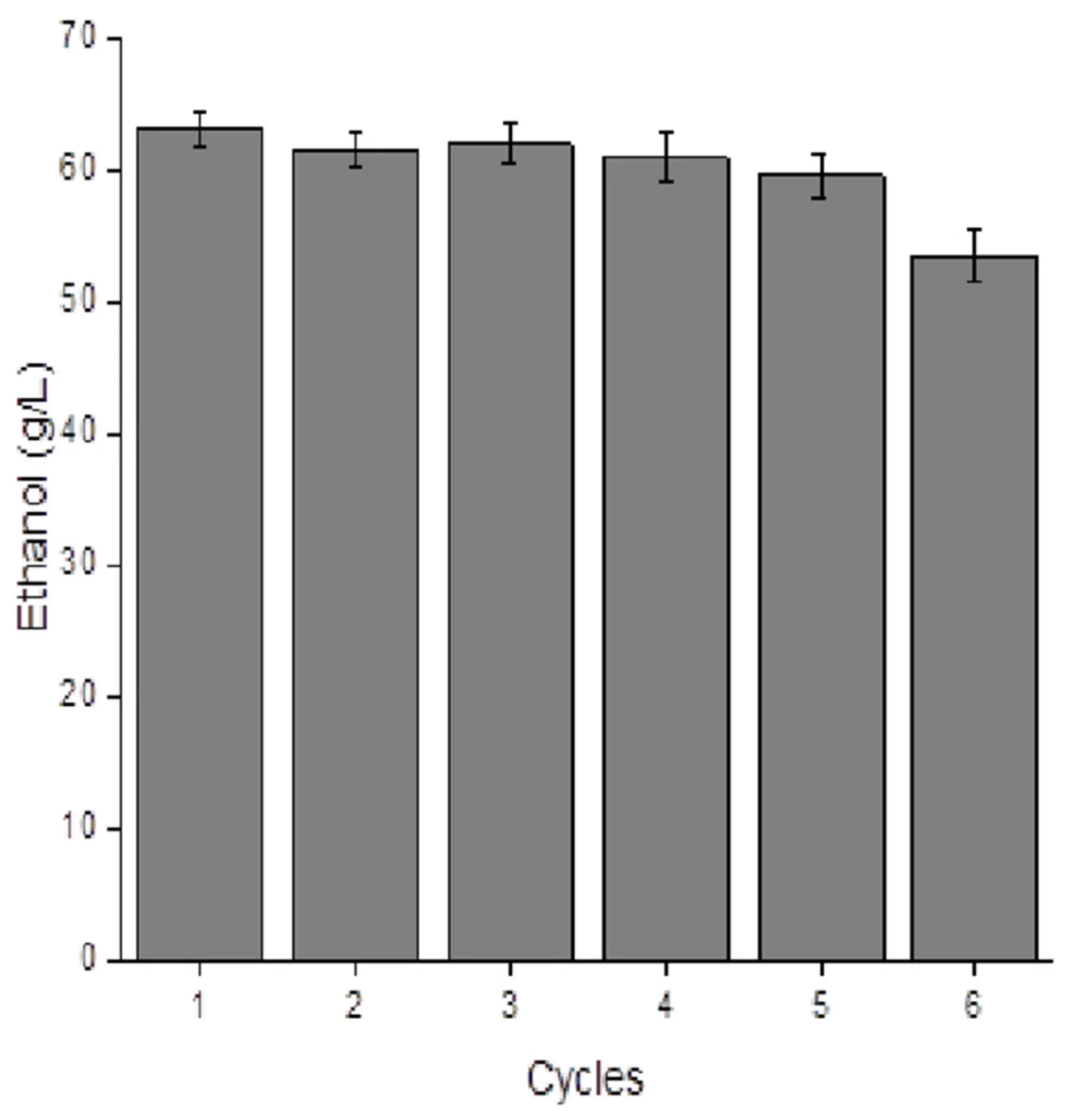
Reuse cycles of co-culture of *Saccharomyces cerevisiae* and *Kluyveromyces marxianus* immobilized on chitosan-coated manganese ferrite nanoparticles during sugarcane molasses fermentation at 20 °Brix and 32 °C for 42 h. Ethanol production was evaluated during successive fermentation cycles after magnetic recovery of the immobilized biomass. Values represent mean ± standard deviation of triplicate experiments.

The reuse assays showed that the immobilized co-culture of *Saccharomyces cerevisiae* and *Kluyveromyces marxianus* performed well across consecutive fermentation cycles, indicating biocatalytic system performance (Figure 5). Although direct measurements of viable cell count, metabolic activity, or biomass retention were not performed, the maintenance of fermentation performance and ethanol production across repeated fermentation cycles suggests operational stability of the immobilized system under the conditions evaluated. Nevertheless, future studies including CFU determination, live/dead staining, and biomass retention analyses are required to directly assess cell viability and physiological status during reuse.

Ethanol production was relatively stable over the first five cycles of reuse, indicating that the immobilized biomass maintained metabolic activity and viability after serial magnetic recovery. These findings suggest that chitosan-coated manganese ferrite nanoparticles served as a stable support, maintaining living yeast cells during subsequent fermentation events. Several effects related to immobilization may be responsible for the stability we noticed in the first cycles. Undoubtedly, the chitosan coating enhanced electrostatic interactions and hydrogen bonding between cell walls and the magnetic support, thereby facilitating recovery and reducing biomass loss [15]. Moreover, under typical environmental stresses such as osmotic pressure, ethanol toxicity, and changes in feed supply, immobilization may also enable microbial cells to resist these stresses [11]. Recurring ethanol production showed relative stability after some use, indicating that the immobilization matrix provided a suitable microenvironmental condition for continued cellular activity. A small decrease in ethanol production, which occurred approximately after the fourth and fifth cycles, and in the sixth cycle (Figure 5), may potentially reflect the process of cell leakage, partial inactivation of the metabolically active cells, or the structural alteration in the immobilization matrix after the cell was allowed to be used multiple times. Higher concentrations of toxic metabolites, nutrient losses, and prolonged exposure to ethanol also may negatively influence fermentation activity in subsequent rotations [15]. Moreover, prolonged magnetic separation and agitation may reduce cell-support interactions and partially separate biomass from the nanoparticles [11]. Although a decrease was observed at the end of the cycle, the immobilized co-culture retained a high percentage of its ethanol yield, demonstrating the reliability of the magnetic immobilization system. This behavior has also been observed in repurposed immobilized yeast biocatalysts, whose activity decreases after repeated cycling due to physiological stress and partial biomass removal, yet their fermentative performance is generally better than that of non-immobilized-cell systems [15].

The ability of immobilized yeast biomass to be efficiently recovered and reused is an important technological advantage for industrial bioethanol production. Magnetic separation is a simple and rapid recovery method; thus, there is no need for centrifugation or filtration, thereby reducing both operational complexity and process costs [15,18]. The use of co-cultivation alongside magnetic immobilization could extend process lifespan and reduce inoculum preparation requirements across processing stages, thereby creating sustainable processes. Hence, the results obtained in the present study support the potential use of chitosan-coated manganese ferrite nanoparticles in reusable fermentation systems, offering increased durability and ethanol yield and serving as a useful nanostructure for this methodology.

Future studies should incorporate direct measurements of cell viability, colony-forming units (CFU), and metabolic activity during successive reuse cycles to better understand the physiological behavior and long-term stability of immobilized yeast populations.

### 4.4. Volatile Compound Production in Different Fermentation Systems

The production of volatile compounds during alcoholic fermentation is heavily dependent on yeast metabolism, fermentation circumstances, and the interplay of microbial species [23]. The results of the present study show significant differences in the production of volatile metabolites among monoculture, co-culture, non-immobilized-cell, and immobilized fermentation systems (Table 3) and reveal that both co-culture and magnetic immobilization significantly affected the aromatic profile of the fermented medium. Compared with *S. cerevisiae*, *K. marxianus* showed lower ethanol concentrations but higher ethyl acetate and higher alcohol levels (Table 3). The latter behavior is consistent with the metabolic profiles of *K. marxianus*, which are characterized by high levels of aroma-active metabolites, including esters and fusel alcohols [1]. Increased biosynthesis of higher alcohols and ethyl acetate may also be associated with its enzymatic catabolism of the active amino acids in the Ehrlich pathway and rapid sugar metabolism [1]. While high levels may, in some cases, adversely affect the drink’s quality, low concentrations could be useful for enhancing the complexity of aroma and sensory features [3].

In a separate process, *S. cerevisiae* 150 resulted in higher ethanol levels and lower levels of secondary volatile compounds (Table 3), indicating that sugar is converted more efficiently into ethanol. *S. cerevisiae* is well-known for its high fermentative capability and ethanol tolerance, properties that promote carbon flux toward ethanol production instead of secondary metabolite synthesis [2]. The relatively low concentrations of acetaldehyde and methanol in this system are also desirable, as these compounds are usually considered undesirable at high concentrations due to their adverse sensory and toxicological properties.

The addition of *S. cerevisiae* 150 to chitosan-coated magnetic nanoparticles resulted in a small increase in ethanol and overall alcohol production, with no significant accumulation of unwanted compounds during immobilization (Table 3). Thus, immobilization enhanced fermentative efficiency and preserved stable metabolic regulation. Immobilization has been proposed to increase local cell density and improve metabolic activity by creating protective microenvironments that enhance resilience to ethanol toxicity and osmotic stress, thereby promoting metabolic hyperactivation [21]. In addition, chitosan coating may enhance cell viability and stability during fermentation [15]. Differences in volatile metabolite production among monoculture, co-culture, free-cell, and immobilized fermentations suggest that both microbial interactions and immobilization conditions influence secondary metabolism [21]. Immobilization may modify local cellular microenvironments and mass-transfer conditions, thereby affecting metabolic fluxes associated with the biosynthesis of aroma compounds. Consequently, the observed changes in volatile profiles may reflect the combined effects of yeast–yeast interactions and the cells’ immobilized state.

The co-culture strategies had even better fermentation performance and produced more complicated volatile profiles than monocultures [1,3]. Between the *K. marxianus* and *S. cerevisiae* 150, the production of ethanol, higher alcohol, and acetaldehyde rose considerably, indicating a strong metabolic interaction between the yeast species (Table 3). Those synergistic effects may be due to complementary substrate utilization patterns, cross-feeding mechanisms, and differences in enzyme activities between the two yeasts. *K. marxianus* is effective at swiftly absorbing nutrients and breaking them down into metabolites that stimulate *S. cerevisiae* metabolism, and *S. cerevisiae* brings along high ethanol productivity and tolerance to stress fermentation conditions. The immobilized co-culture system was reported to perform best overall, producing the highest ethanol yield, as well as increased ethyl acetate and higher alcohols [1,3,11]. The enhanced production of aroma compounds under immobilized conditions indicates that immobilization improved fermentation efficiency and altered metabolic pathways associated with secondary metabolite biosynthesis. Immobilization could promote both closer proximity among yeast species and stronger metabolic cooperation, as well as improved exchange of metabolites relevant to ester and higher alcohol biosynthesis [1,21]. The lower concentrations of acetaldehyde in the immobilized product (compared with the non-immobilized co-culture) indicate that immobilization may stabilize the redox equilibrium and enhance the convertibility of intermediate metabolites during fermentation [1,21]. Broadly speaking, co-culture and magnetic immobilization can both considerably modify the metabolic activity of yeasts during alcoholic fermentation. Such approaches not only increase ethanol production but also alter the composition of volatile metabolites; therefore, they may contribute to improved sensory and aromatic properties in fermented goods. Therefore, immobilized mixed cultures of *S. cerevisiae* and *K. marxianus* can be considered for the development of a suitable, reusable biobased yeast system to achieve bioethanol fermentation and to produce fermented products with better aromatic quality. The differences observed in volatile metabolite production among monoculture, co-culture, free-cell, and immobilized fermentations indicate that both microbial interactions and immobilization conditions influenced secondary metabolism. Co-cultivation may alter the availability of metabolic intermediates and modify yeast physiological responses, leading to changes in the synthesis of aroma-related compounds. Furthermore, immobilization on magnetic nanoparticles can affect mass-transfer phenomena and local cellular microenvironments, which may contribute to the distinct volatile profiles detected in the fermented products.

Volatile compounds in alcoholic beverages, including fermentation products such as esters, higher alcohols, aldehydes, and acetals, influence aroma, flavor complexity, and sensory quality, especially in beverages like rum [1]. More volatile compounds, such as ethyl acetate, produce fruity, pleasant aromatic notes at moderate concentrations, while higher alcohols contribute to the body and complexity of distilled beverages [1]. The generation of such metabolites is strongly affected by yeast species, fermentation conditions, nutrient availability, and microbial interactions. The distinct volatile profiles reported for co-culture and immobilized systems (Table 3) suggest they may have applications not only in enhancing ethanol production but also in modulating the sensory profile of fermented and distilled products from sugarcane molasses. The increased levels of volatile compounds in the mixed cultures, especially under immobilized conditions, may support rum production, as controlled concentrations of esters and higher alcohols are considered important constituents of the typical aroma of sugarcane distillates [1]. The higher concentrations of ethyl acetate and higher alcohols observed in the co-culture systems suggest that metabolic interactions between *Saccharomyces cerevisiae* and *Kluyveromyces marxianus* enhanced the formation of aroma-related compounds (Table 3). Furthermore, immobilization may have helped maintain metabolic activity and cellular proximity, potentially affecting precursor availability and the metabolic fluxes involved in volatile compound biosynthesis [11,16,21], these effects could explain the distinct volatile profiles observed between immobilized and free-cell fermentation systems.

Yet an overabundance of some compounds, especially aldehydes or methanol, has adverse impacts on drinkability and safety. Although the systems differed in this study, all measured concentrations remained below the permissible limits set by the Mexican standard PROY-NOM-199-SCFI-2015 for higher alcohols, methanol, aldehydes, and esters. The results of this study show, for instance, that the immobilized co-culture systems not only increased fermentative efficiency and ethanol productivity but also generated volatile compound profiles compatible with distilled alcoholic beverage regulatory criteria. Thus, the dual co-cultivation–magnetic immobilization combination could be a promising technological strategy for developing fermentation processes to brew a wide range of ethanol beverages made from sugarcane, with high aromatic complexity, stability, and reliability in meeting quality standards. Although additional parameters such as specific surface area, zeta potential, particle size distribution, and colloidal stability were not evaluated in the present work, future studies that address these properties may contribute to a more detailed understanding of the interactions between immobilized yeast cells and magnetic nanostructured supports and their influence on fermentation performance.

From an industrial perspective, the use of magnetic nanoparticle-assisted immobilization offers several potential advantages, including simplified biomass recovery, reduced reliance on centrifugation or filtration, and the possibility of repeated biomass reuse. These characteristics may contribute to lower operational costs and improved process sustainability in industrial fermentation systems. Furthermore, the enhanced ethanol productivity observed in the immobilized co-culture suggests potential benefits for large-scale bioethanol production and other fermentation-based bioprocesses.

Nevertheless, several limitations should be considered. The present study was conducted at a laboratory scale, and direct assessments of cell viability, immobilization reproducibility across batches, and long-term support stability were not performed. In addition, further studies evaluating process economics, large-scale operational performance, and additional physicochemical characterization of the support material are required before industrial implementation can be fully assessed.

## 5. Conclusions

In conclusion, this study demonstrated that chitosan-coated manganese ferrite nanoparticles constitute an efficient nanostructured platform for the co-immobilization of *Saccharomyces cerevisiae* and *Kluyveromyces marxianus* during alcoholic fermentation of sugarcane-derived substrates. The magnetic co-immobilization strategy improved fermentation performance compared with non-immobilized-cell systems, as evidenced by enhanced sugar utilization, higher ethanol productivity, and successful recovery and repeated reuse of the immobilized biocatalytic system during fermentation cycles. In addition, the immobilized co-culture exhibited changes in volatile metabolite profiles, indicating that immobilization and yeast–yeast interactions influenced fermentation-derived metabolites. Overall, the results support the potential of chitosan-coated manganese ferrite nanoparticles as reusable nanobiocatalytic supports for the development of more efficient fermentation processes. Further studies, including direct measurements of cell viability, biomass retention, and metabolic activity, are required to better understand the physiological behavior of immobilized cells during long-term reuse.

## Figures and Tables

**Table 1:** Characterization of molasses and sugarcane juice.

Parameters	Unit	Molasses	Sugarcane Juice
Soluble solids	°Brix	82.16 ± 1.16	17.51 ± 0.08
pH		5.23 ± 0.01	3.51 ± 0.07
Fermentable sugars	(%)	66.34 ± 1.14	25.03 ± 1.12
Ashes	(%)	9.17 ± 0.09	0.19 ± 0.04
Nitrogen	(%)	0.21 ± 0.06	0

**Table 2:** Minerals present in molasses and sugarcane juice ashes.

Minerals (%)	Molasses	Sugarcane Juice
Magnesium	0.01	0
Phosphorus	0.01	0.01
Potassium	0.09	0.03
Calcium	0.03	0.03
Sulfur	0.01	0.01

**Table 3:** Comparison of major volatile compound production (g/100 L) in fermented molasses-containing media (ND = not detectable). (Different letters within a column indicate significant differences (*p* < 0.01)).

Yeast System	Acetaldehyde (g/100 L)	Ethyl Acetate (g/100 L)	Acetal (g/100 L)	Methanol (g/100 L)	1-Butanol (g/100 L)	Higher Alcohols (g/100 L)	Ethanol (g/L)
*K. marxianus*	1.02 ± 0.13 ^b^	3.54 ± 1.01 ^b^	2.21 ± 3.43 ^b^	1.3 ± 1.02 ^a^	ND	145.0± 1.0 ^c^	29.1 ± 1.2 ^d^
*S. cerevisiae* 150	ND	0.82 ± 0.15 ^d^	ND	0.15 ± 0.08 ^b^	ND	78.05 ± 0.7 ^d^	50.7 ± 3.1 ^c^
*S. cerevisiae* 150 with C-MNP	ND	0.86 ± 0.55 ^d^	ND	0.11 ± 0.11 ^b^	ND	83.02 ± 0.7 ^d^	56.1 ± 4.0 ^c^
*K. marxianus* and *S. cerevisiae* 150	15.37 ± 0.48 ^a^	1.36 ± 0.05 ^c^	6.31 ± 1.98 ^a^	1.53 ± 1.02 ^a^	1.09 ± 0.37 ^a^	180.0 ± 1.0 ^b^	63.1 ± 2.5 ^b^
*K. marxianus* and *S. cerevisiae* 150 with C-MNP	3.66 ± 2.11 ^b^	4.77 ± 0.18 ^a^	2.25 ± 1.56 ^b^	1.61 ± 0.42 ^a^	0.98 ± 0.09 ^a^	185.4± 1.2 ^a^	70.0 ± 3.7 ^a^

## Data Availability

The original contributions presented in this study are included in the article. Further inquiries can be directed to the corresponding authors.
